# Subnational trends and inequalities of under-immunisation and zero-dose among children aged 12–23 months in Uganda: a national population-based cross-sectional study

**DOI:** 10.1136/bmjopen-2024-093619

**Published:** 2025-01-15

**Authors:** Ronald Wasswa, Rornald Muhumuza Kananura, Peter Waiswa, Jennifer Harris Requejo, Thiago M Santos, Aluisio J D Barros

**Affiliations:** 1 Department of Health Policy Planning and Management, Makerere University School of Public Health, Kampala, Uganda; 2 Makerere University Center of Excellence for Maternal and Newborn Health, Kampala, Uganda; 3 African Population and Health Research Center (APHRC), Nairobi, Kenya; 4 Global Public Health, Karolinska Institutet, Stockholm, Sweden; 5 Busoga Health Forum, Jinja, Uganda; 6 Global Financing Facility for Women, Children, and Adolescents, World Bank, Washington, DC, USA; 7 Department of International Health, Johns Hopkins University, Baltimore, Maryland, USA; 8 International Center for Equity in Health, Universidade Federal de Pelotas, Pelotas, Brazil

**Keywords:** Immunisation Programmes, Health Equity, Vaccination, Child

## Abstract

**Abstract:**

**Objective:**

Despite the Global Vaccine Action Plan’s goal of at least 90% vaccine coverage for all children, Uganda has made limited progress in vaccination over the past decade. The objective of this study was to examine the subnational trends in the prevalence and inequalities in under-immunisation and zero-dose among children aged 12–23 months in Uganda.

**Study design:**

A retrospective national cross-sectional study.

**Setting:**

Uganda

**Participants:**

Uganda Demographic and Health Survey secondary data of only children aged 12–23 months. The samples selected for analyses were 1507 in 2006, 1409 in 2011 and 2650 children in 2016.

**Outcome measure:**

The primary outcomes were under-immunisation and zero-dose vaccination.

Absolute and relative inequality measures were used in the analysis.

**Results:**

From 2006 to 2016, the under-vaccination rate decreased by 21%, but remained high at 40.8%. The zero-dose vaccination rate dropped by 82%, affecting 1.2% of children in 2016. Subnational inequalities in under-vaccination increased over time with widening gaps between regions. While inequalities across wealth quintiles, maternal education levels and places of residence narrowed, children of mothers with lower education levels continued to have the highest under-vaccination rates. The rural–urban gap for zero-dose vaccination remained unchanged, with rural children disproportionately impacted.

**Conclusion:**

While some progress was made in reducing under-vaccination rates in Uganda within the study period, no region achieved an under-vaccination rate below 20%. This indicates significant challenges in reaching the Sustainable Development Goal target of at least 80% immunisation coverage. Targeted interventions are necessary to improve healthcare access, enhance public health communication and strengthen the health system, particularly in underserved communities and among vulnerable populations.

**STRENGTHS AND LIMITATIONS OF THIS STUDY**

- The study uses data from three reliable and nationally representative Uganda Demographic and Health Surveys.
- It examines subnational inequalities in under-immunisation and zero-dose vaccination, revealing disparities masked by national averages.
- It employs both absolute and relative inequality measures for a comprehensive regional and socioeconomic assessment.
- We also acknowledge potential recall bias that may have affected the accuracy of vaccination data due to reliance on mothers’ memory in the absence of the vaccination card.
- The most recent available data is from 2016, which may not fully reflect the current situation, and the cross-sectional design limits the ability to draw causal inferences.

## Introduction

 Immunisation is one of the most powerful and cost-effective interventions for preventing diseases.^1^,^6^ Over a span of 20 years, vaccines have averted 36 million deaths of children under 5 in low- and middle-income countries, with an additional 28 million projected to be averted by 2030.^7^ Recognising these benefits, the World Health Assembly endorsed the Global Vaccine Action Plan (GVAP), aiming for universal access to immunisation for every child by 2030.^8^ The GVAP set targets of at least 90% coverage at the national level, with no subnational region with a coverage of less than 80% for all routine immunisation vaccines by 2030.^8^,^10^ Similarly, the Immunization Agenda 2030 also aims to ensure full immunisation for every child, regardless of location, age, socioeconomic status or gender-related barriers.^8^,^10^

Despite tremendous progress in vaccination coverage, a significant proportion of children remain unvaccinated or under-vaccinated, particularly in sub-Saharan Africa. For instance, in 2022, there were an estimated 14.3 million unvaccinated children (zero-dose children), with an additional 6.2 million partially vaccinated, the majority of whom reside in sub-Saharan Africa.^11^ In Uganda, national coverage for full basic vaccination among children aged 12–23 months has seen slow progress over the past decade, reaching 55% in 2016.^12^ Regional inequalities in routine vaccination coverage persist despite initiatives like the Reaching Every District initiative introduced in 2004 to increase coverage and equity in immunisation across communities.^13^,^15^ Despite efforts from programmes like the Uganda National Expanded Program on Immunization and the Maternal and Child Health Integrated Program, Uganda still has a substantial number of zero-dose children.^12^

Detailed subnational trends in immunisation coverage remain unclear in Uganda due to changes in the subregional groupings in the successive demographic health surveys. This lack of clarity in subregions limits efforts to address regional inequities effectively. While studies in sub-Saharan Africa, including Uganda, have examined factors associated with immunisation coverage, limited information exists on subnational inequalities in Uganda to inform targeted interventions.^16^,^24^ Therefore, this study examined subnational trends in the prevalence and inequalities in under-immunisation and zero-dose among children aged 12–23 months in Uganda. By understanding these trends and disparities, targeted interventions can be developed to improve immunisation coverage and reduce regional inequalities.

## Methods

### Data source, acquisition and population

This study used data from three consecutive Uganda Demographic and Health Surveys (UDHSs) conducted in 2006, 2011 and 2016. Approval to download and use these deidentified datasets was obtained from the Measure DHS website (www.measuredhs.com) on request, ensuring ethical compliance for secondary data use.^25^ Each UDHS is nationally representative and employs a two-stage cluster sampling design. In the first stage, clusters are selected from a national sampling frame, while in the second stage, households are systematically sampled within each cluster. Detailed information on the sampling methodology and the standardised tools can be found in the UDHS final reports.^12 26 27^

For this study, we focused on children aged 12–23 months, including 1507, 1409 and 2650 children in 2006, 2011, and 2016, respectively. The UDHS collects data at both household and individual levels, covering a wide range of health and demographic indicators, including immunisation, maternal and child health and nutrition.

### Data curation

On obtaining the datasets^25^ we conducted a thorough data-curation process to ensure readiness for analysis. This involved extracting and filtering data specifically for children aged 12–23 months and immunisation indicators, along with additional variables on household and maternal characteristics. We addressed missing or implausible values through data cleaning. To ensure consistency and comparability across survey years, all study variables were standardised. A data dictionary was generated to document all transformations, recoding decisions and data-cleaning steps.

### Harmonisation of subnational boundaries

Demographic health surveys serve as a crucial tool for providing reliable estimates of survey indicators at both the national and subnational levels.^12 26 27^ However, Uganda has experienced changes in the number of subregions in each successive survey, which limits their comparability over time. With the pressing need to achieve the Sustainable Development Goals (SDGs), there is a growing demand to understand subnational patterns for informed decision-making and efficient programme implementation. To address this, we reconstructed the subregions in the 2016 and 2011 surveys to align with the nine subregions included in 2006. This alignment was accomplished using a methodology^28 29^ that leveraged the Integrated Public Use Microdata Series (IPUMS)- International geographical data files^30^ and the DHS Spatial Repository^31^ to harmonise the subregional boundaries across surveys. The IPUMS-DHS project specifically supports harmonisation by providing consistently coded variables, cross-survey documentation and customisable datasets that enhance comparability over time. Our approach included assigning a standardised identifier to each harmonised subregion, checking the consistency of area boundaries across surveys and ensuring that the geographical structure aligns with the 2006 baseline. This process enables reliable trend analysis at the subnational level. Additional details of the harmonisation process have been documented elsewhere.^28 29 32^

### Selection and measurement of variables

#### Outcomes

Our study focused on two key indicators: under-vaccination and zero-dose vaccination, which were estimated from data collected through interviews with mothers regarding their children’s vaccination status. Under-vaccination was defined as a child not receiving all the following vaccine doses: one dose of BCG, three doses of polio, three doses of pentavalent and one dose of a measles-containing vaccine. Zero-dose was defined as a child not receiving a single dose of any of the above vaccines. The child’s vaccination card is the primary source of data collected through household surveys on the vaccines administered and the number of doses. If a vaccination card is unavailable, survey interviewers ask caretakers/mothers about their children’s vaccination history, the vaccines administered and the number of doses received. When mothers could not recall their child’s vaccination status, the child was categorised as non-vaccinated for that specific vaccine.

#### Equity stratifiers

Four key equity stratifiers were used to disaggregate under-vaccination and zero-dose vaccination: subnational regions, maternal education level, wealth quintiles and place of residence. These variables were selected based on their significance in prior research^33 34^ and their inclusion in the WHO Health Inequality Monitoring Handbook.^35^ The UDHS employs principal component analyses on various household assets, dwelling characteristics and access to basic amenities to generate a wealth index, which is categorised into five equally sized groups, the quintiles, ranging from poorest to richest.^36^ Maternal education levels were classified as none, primary and secondary or higher, while place of residence was categorised as urban or rural. Additionally, the nine harmonised subnational regions were used.

### Statistical analysis

The data were weighted using the sample weight variable from the DHS to ensure representativeness, and the weighting variable was applied in all statistical analyses.^12 26 27^ In the analysis, we began by disaggregating the proportions of under-vaccination and zero-dose vaccination across the four equity stratifiers. Subsequently, four inequality summary measures were employed, detailed in table 1. These measures included both absolute and relative metrics, such as absolute difference, weighted mean absolute difference from the overall mean, ratio and weighted mean ratio from the overall mean. This methodological approach aligns with previous studies.^37 38^

**Table 1:** Selected summary measures of inequality in under-vaccination rates

Summary measure	Measure type	Description	Formula
Difference	Simple measure of absolute inequality	The difference between the indicator value for a category ($y_{high}$) with the highest under-vaccination rate, and the value of the category ($y_{low}$), with the lowest under-vaccination rate	$y_{high}-y_{low}$
Weighted mean absolute difference from the overall mean (WMADM)	Complex measure of absolute inequality	The weighted average of the difference between the under-vaccination rate for subregion *j* ($y_{j}$), and the national average (μ), Differences are weighted by each subregion’s share of the total population (${pop}_{j}$),	$\frac{\sum_{j}pop_{j}\times \left|y_{j}-\mu \right|}{pop}$
Ratio	Simple measure of relative inequality	The ratio of the category with the highest under-vaccination rate ($y_{\left(high\right)}$) to the category with the lowest undervaccination rate ($y_{\left(low\right)}$).	$\frac{y_{\left(high\right)}}{y_{\left(low\right)}}$
Weighted mean relative difference from the overall mean (WMRDM)	Complex measure of relative inequality	The weighted average of the difference between the indicator value for subregion *j* (γ*j*) and the national average (*μ*) divided by the national average (*μ*) and multiplied by 100. Differences are weighted by each subregion’s share of the total population (*pj*)	$\frac{WMADM}{\mu }\times 100$

Hosseinpoor and colleagues^37^ highlighted that simple measures of inequality, such as differences and ratios, often yield findings comparable to those of more complex measures. In our analysis, differences and ratios, effectively revealed disparities between the best-performing and worst-performing subregions, while complex measures provided additional details by accounting for variations between each subregion and the national average, weighted by population size. This similarity between simple and complex measures strengthens the validity of our results and highlights the urgency of addressing the identified inequities and gaps in health coverage.

### Patient and public involvement

As this study used secondary data, there was no direct involvement of patients or the public in the study design, data collection or analysis processes.

## Results

Table 2 presents the distribution of the study population across selected equity stratifiers for the years 2006, 2011 and 2016. The household wealth index shows a consistent representation across all categories each comprising roughly 14.0–24.0% of the population. Maternal education levels indicate that the majority of the children had mothers with at least primary education (61.4–65.7%), followed by secondary or higher education (13.7–29.4%). Results also indicate that children in urban areas constituted 9.6–22.7% of the child population, while rural children ranged between 77.3–90.4% for the entire study period. The distribution of children across regions is fairly even, with the largest population shares in the Eastern (17.0–17.4%) and Western (13.1–15.1%) regions.

**Table 2:** Distribution of the study population by the selected stratifiers

Equity stratifier	2006 (n=1507)n (%)	2011 (n=1409)n (%)	2016 (n=2650)n (%)
**Wealth index**			
Lowest	320 (21.2)	317 (22.5)	586 (22.1)
Second	360 (23.9)	309 (21.9)	577 (21.8)
Middle	331 (22.0)	258 (18.3)	497 (18.7)
Higher	285 (18.9)	261 (18.5)	457 (17.2)
Highest	211 (14.0)	264 (18.7)	534 (20.2)
**Maternal education**			
No education	310 (20.6)	184 (13.1)	242 (9.1)
Primary	990 (65.7)	892 (63.3)	1628 (61.4)
Secondary or higher	207 (13.7)	332 (23.6)	780 (29.4)
**Place of residence**			
Urban	144 (9.6)	189 (13.4)	602 (22.7)
Rural	1363 (90.4)	1220 (86.6)	2049 (77.3)
**Region**			
South Buganda	150 (9.9)	146 (10.3)	329 (12.4)
North Buganda	123 (8.2)	156 (11.1)	282 (10.6)
Kampala	66 (4.3)	78 (5.6)	122 (4.6)
Busoga	174 (11.6)	162 (11.5)	276 (10.4)
Eastern	256 (17.0)	244 (17.3)	462 (17.4)
North	250 (16.6)	197 (14.0)	339 (12.8)
West Nile	81 (5.3)	74 (5.3)	195 (7.4)
Western	228 (15.1)	184 (13.1)	366 (13.8)
Southwest	181 (12.0)	168 (11.9)	280 (10.6)

n is the frequency; % is the percentage; all results based on weighted data.

**Figure 1:**
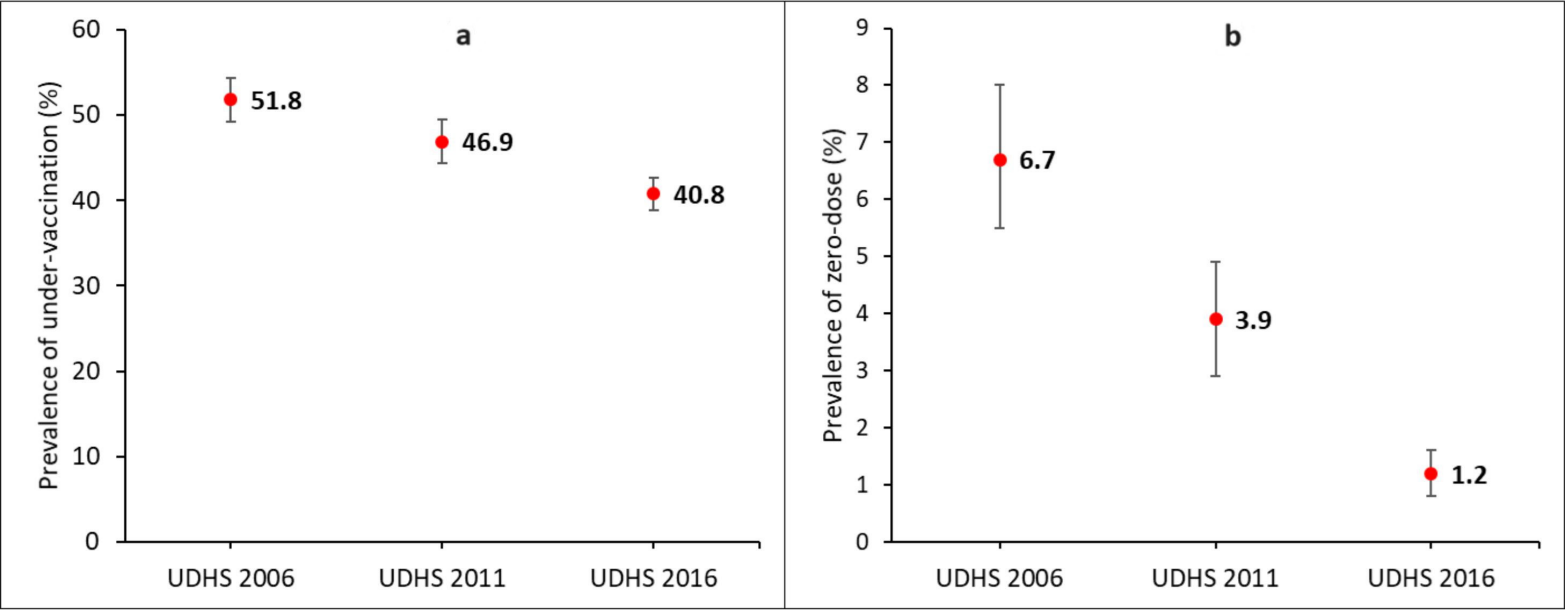
National trends in under-vaccination (**a**), and zero-dose vaccination (**b**) in Uganda. Bars represent 95% CI.

Figure 1 shows the trends in under-vaccination and zero-dose vaccination for the period of 10 years. The prevalence of undervaccinated children reduced by 21%, from 51.8% in 2006 to 40.8% in 2016. In the same period, the percentage of zero-dose children reduced by 82%, from 6.7% to 1.2%.

**Figure 2:**
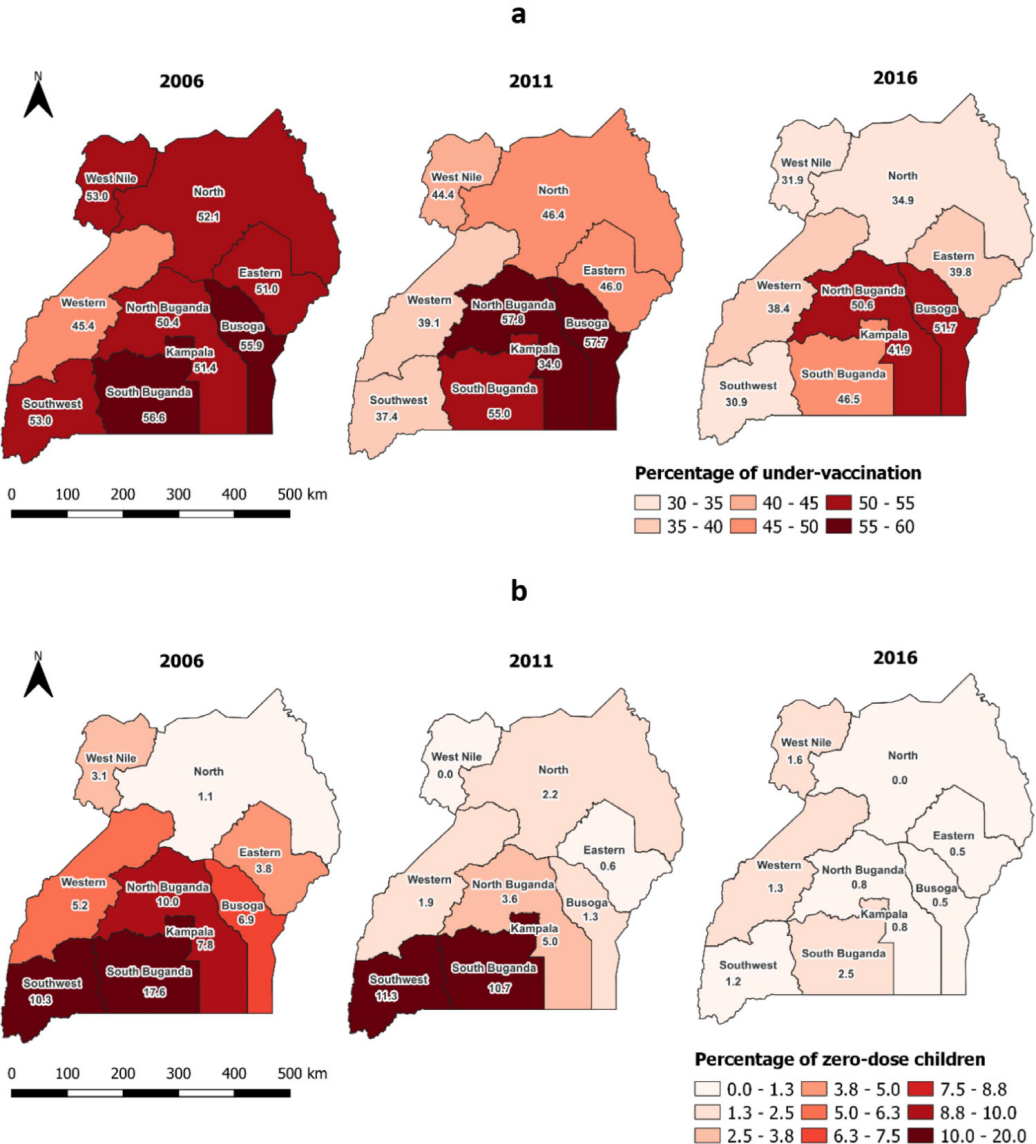
Subnational trends in under-vaccination (**a**) and zero-dose children (**b**) in Uganda.

The trends in under-vaccination rates for children (figure 2) show a significant improvement across subregions from 2006 to 2016. The Southwest region experienced the largest reduction, with a 22 percentage point decrease, followed closely by West Nile with a 21 percentage point reduction. The Busoga and Western regions experienced the smallest decline of 7 and 4 percentage points, respectively. In contrast, the North Buganda region’s under-vaccination rate remained stagnant.

Results also show a substantial decrease in the prevalence of zero-dose children across subregions. The South Buganda region had the most notable improvement, with a decline from 17.6% in 2006 to 2.5% in 2016. This was followed by North Buganda and Southwest region with each experiencing a decline of 9 percentage points (figure 2).

**Figure 3:**
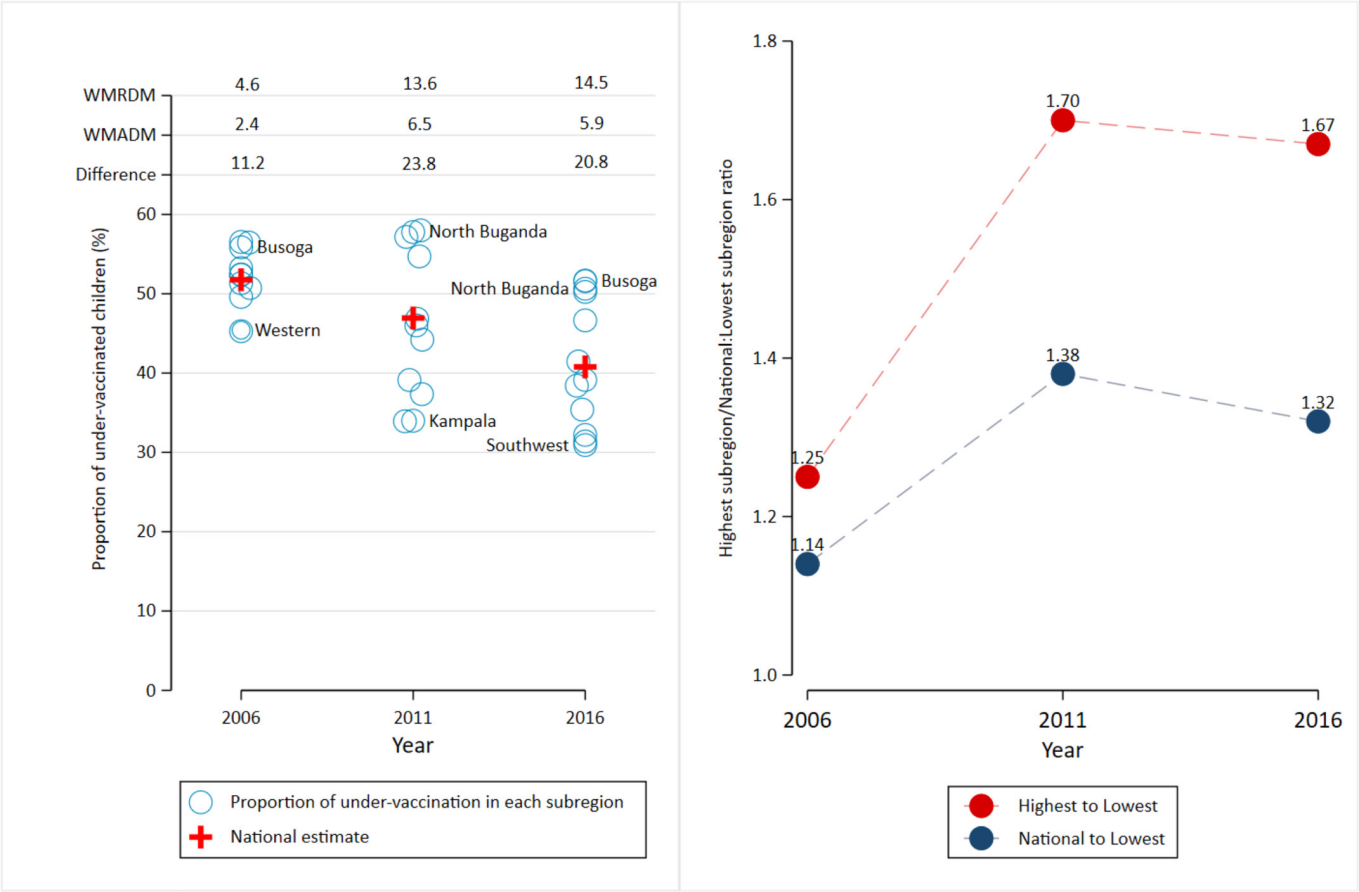
Subnational inequality gap in the prevalence of under-vaccination. WMADM, weighted mean absolute difference from the overall mean; WMRDM, weighted mean relative difference from the overall mean.

However, despite these positive changes, there was a significant increase in subnational inequality in under-vaccination rates. The weighted mean relative difference in weighted mean ratio from the overall mean rose from 4.6 in 2006 to 14.5 in 2016, accompanied by an increase in the ratio and weighted mean absolute difference from the overall mean (figure 3). The gap between the national prevalence and the prevalence in the best-performing region also widened, increasing from 1.14 in 2006 to 1.32 in 2016 (figure 3).

**Figure 4:**
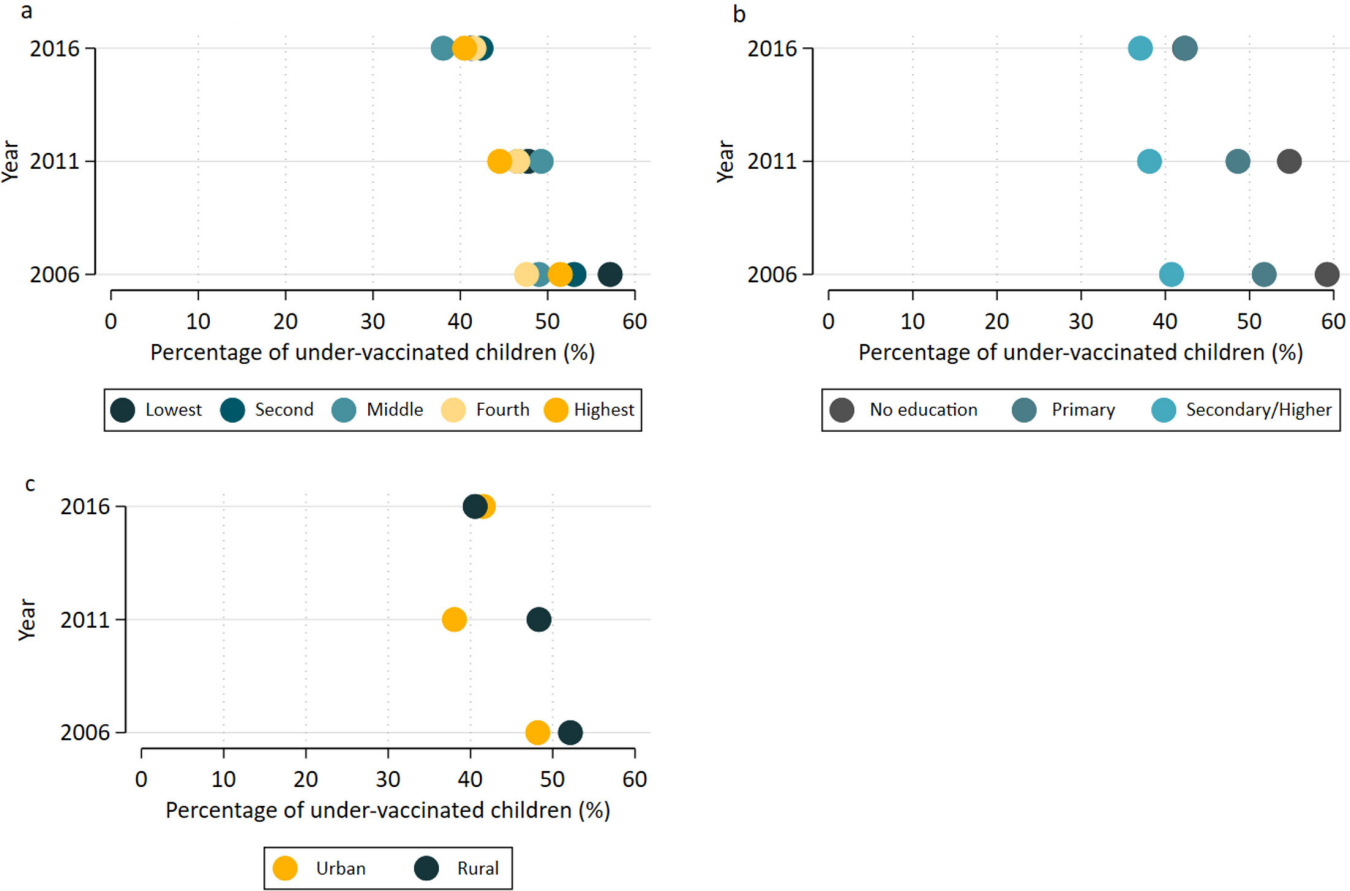
Inequality in the percentage of under-vaccination: by wealth (**a**), maternal education (**b**), and place of residence (**c**).

Results further revealed that inequality in under-vaccination rates also varied by place of residence, maternal education and wealth index from 2006 to 2016. In relation to household wealth, the prevalence of under-immunisation decreased across all quintiles. In 2006, undervaccination among the poorest was 57.2%, falling to 41.3% by 2016, and for the richest, it decreased from 51.4% to 40.5% (figure 4). In addition, the absolute difference in wealth index inequality reduced from 9.4 percentage points in 2006 to 4.4 percentage points in 2016 (online supplemental file 1).

Children of mothers with no education or primary education consistently had higher under-vaccination rates compared with those with secondary or higher education levels. In 2006, under-vaccination for children of mothers with no education was 59.2%, dropping to 42.3% by 2016, while for those with secondary or higher education, under-vaccination decreased from 40.7% to 37.0% (figure 4). In addition, the absolute difference sharply dropped from 18.5 percentage points in 2006 to 5.4 percentage points in 2016 (online supplemental file 1).

The prevalence of under-vaccination in rural areas decreased from 52.2% to 40.6%, and in urban areas from 48.2% to 41.6% by 2016. Place of residence inequality also diminished, with the absolute difference between urban and rural areas dropping from 4.0 percentage points in 2006 to 1.0 percentage point in 2016 (online supplemental file 1). This suggests a narrowing rural–urban inequality gap despite the persistently high prevalence of undervaccination.

Findings also showed that from 2006 to 2016, the prevalence of zero-dose children consistently declined across all stratifiers, despite persistent inequalities among the less privileged (online supplemental file 1). Among the poorest households, the zero-dose rate dropped from 3.8% to 1.4%, while among the richest households, it declined from 7.3% to 0.2%. The lowest prevalence was among children whose mothers had higher education levels, with rates decreasing from 8.0% to 2.6% among mothers with no education, and from 3.5% to 0.3% among those with secondary or higher education. Similarly, the prevalence in rural areas reduced from 6.8% to 1.4%, and in urban areas from 5.8% to 0.5% (online supplemental file 1).

## Discussion

In line with earlier findings^39^ our results indicate that Uganda has experienced a substantial decrease in zero-dose vaccination rates over the study years and a slight reduction in under-vaccination rates across its regions. However, none of the regions achieved an under-vaccination rate below 20% by the end of the study period, indicating challenges in reaching the SDG target of at least 80% immunisation coverage.^8^,^10^ In addition, our findings align with those of Ssebagereka and colleagues^39^ to highlight the significant role of socioeconomic factors and regional disparities in influencing vaccine coverage. We noted a widening subregional inequality, with under-vaccination rates diverging over time.

There is a noticeable disparity between the national prevalence of under-vaccination and the prevalence in the best-performing region, highlighting growing inequities across Uganda between 2006 and 2016. While some regions showed progress in reducing under-vaccination rates, the national rate increased at a faster pace, highlighting a concerning trend where national efforts are not resulting in equal advancements across all regions. Particularly, North Buganda and Busoga exhibit little or stagnant progress over the study decade in regard to under-vaccination. These disparities may stem from differences in socioeconomic status, resource allocation to the health sector and other health system barriers such as a disrupted cold chain, irregular vaccine supplies and distribution, limited healthcare personnel and infrastructure and significant distances between health facilities and communities.^40^,^48^

Consistent with earlier studies conducted in sub-Saharan Africa,^49^,^51^ our study found a significant reduction in rural–urban inequalities in under-immunisation over time. Despite this positive trend, the prevalence of under-vaccination remained high among both urban and rural children, indicating that while disparities have narrowed, overall vaccination coverage still lags behind expectations. However, in contrast to the trend observed for under-immunisation, the rural–urban gap in zero-dose vaccinations did not show a comparable reduction. Rural children continued to bear a disproportionate burden of zero-dose vaccination compared with their urban counterparts. This finding contrasts with Ssebagereka *et al*’s study, which reported a narrowing of urban–rural disparities in zero-dose vaccinations over a similar period.^39^ Several factors may explain this divergence. First, the definition of zero-dose vaccinations slightly differs between the studies: Ssebagereka *et al* define zero-dose as a child who has not received any doses from the national immunisation schedule by 12 months of age, whereas in our study, we considered children who had not received any vaccines within the 12–23 month age range. Additionally, differences in methodology such as the inequality measures employed and our approach to harmonise subnational boundaries across different survey years may have influenced the interpretation of trends over time, contributing to the contrasting findings.

One possible explanation for the persistent inequality in zero-dose vaccinations as noted in this study is the proximity to health facilities. Urban children generally live closer to healthcare services than rural children, making it easier for caretakers to access vaccination services.^52^ We hypothesise that the high prevalence of under-vaccination in urban areas may be significantly influenced by disparities between non-poor and poor urban children. Santos and colleagues revealed that non-poor urban children have an advantage in terms of immunisation compared with their poorer counterparts.^52 53^ In some cases, urban poor children may even have worse immunisation outcomes than rural children.^52^ Addressing these issues requires targeted interventions to improve healthcare access among the urban poor, enhance public health communication about the benefits of vaccination and strengthen the health system to better serve all vulnerable populations.

Disparities based on household wealth significantly decreased by 2016, indicating narrowing inequalities in access to immunisation services among different socioeconomic groups. This was evidenced by the decline in absolute disparity measures, reflecting a narrower gap between wealthy and poor households. While previous studies have shown lower access to healthcare services among the economically disadvantaged,^4041 47 53^,^57^ our findings show a more rapid improvement in immunisation coverage among children in the poorest quintile compared with those in the wealthiest. Uganda ranks highly among the global leaders in implementing pro-equity immunisation efforts, with over 38 strategies addressing both demand and supply challenges.^39 58^ These initiatives have played a significant role in narrowing immunisation equity gaps. Similar approaches have also been shown to reduce disparities in healthcare access and use elsewhere.^59 60^

Our findings showed that children of mothers with lower education levels experience higher rates of under vaccination. This finding aligns with earlier studies, reaffirming the correlation between maternal education and vaccination status among children.^39^,^4147 54 55 61^ Additionally, the inequality gap between the educated and illiterate has narrowed, but the under-vaccination rate for children of mothers with secondary and higher education remained largely unchanged during the study period. While the percentage of educated mothers increased over time, their children’s vaccination rates did not show significant improvement. This suggests that merely increasing maternal education levels may not sufficiently enhance vaccination rates. Implementing targeted outreach programmes for communities with lower maternal education levels, focusing on tailored education, community engagement with local leaders and health workers and strengthening health systems will enhance vaccination access and acceptance.

This study leverages data from three reliable and nationally representative UDHS to examine subnational inequalities in under-immunisation and zero-dose vaccination, revealing disparities often masked by national averages. By employing both absolute and relative inequality measures, it provides a comprehensive assessment across regions and socioeconomic groups. However, limitations include potential recall bias from mothers' reports in the absence of vaccination cards, the use of data from 2016 that may not reflect the current immunisation landscape and the cross-sectional nature of the data which limits causal inferences.

## Conclusion

Despite some progress in reducing under-vaccination rates in Uganda, the country still faces significant challenges in achieving the SDG of 80% immunisation coverage. The analysis reveals widening subregional inequalities, with some regions showing little or no progress in reducing under-vaccination rates. Rural areas continue to bear a disproportionate burden, with urban–rural disparities persisting, particularly in zero-dose vaccinations. Socioeconomic inequalities also persist, with children of lower-income and less-educated mothers being disproportionately affected.

To address these issues, targeted interventions are needed to improve healthcare access, public health communication and health system strengthening, particularly in underserved communities and among vulnerable populations. Additionally, further detailed studies could explore locally important underlying causes of these disparities, while the implementation of evidence-based strategies to optimise vaccine acceptance is evaluated in partnership with local communities, health professionals and authorities. Combining targeted research with collaborative, community-driven solutions can help achieve more equitable vaccination coverage across Uganda.

## supplementary material

10.1136/bmjopen-2024-093619 online supplemental file 1

## Data Availability

Data are available in a public, open access repository.
