# Supplementary material for: Subnational trends and inequalities of under-immunisation and zero-dose among children aged 12–23 months in Uganda: a national population-based cross-sectional study
Source: BMJ Open. 2025 Jan 15;15(1):e093619. doi: 10.1136/bmjopen-2024-093619 (PMC11752051; doi:10.1136/bmjopen-2024-093619)
Supplement: online supplemental file 1 [file bmjopen-15-1-s001.docx]

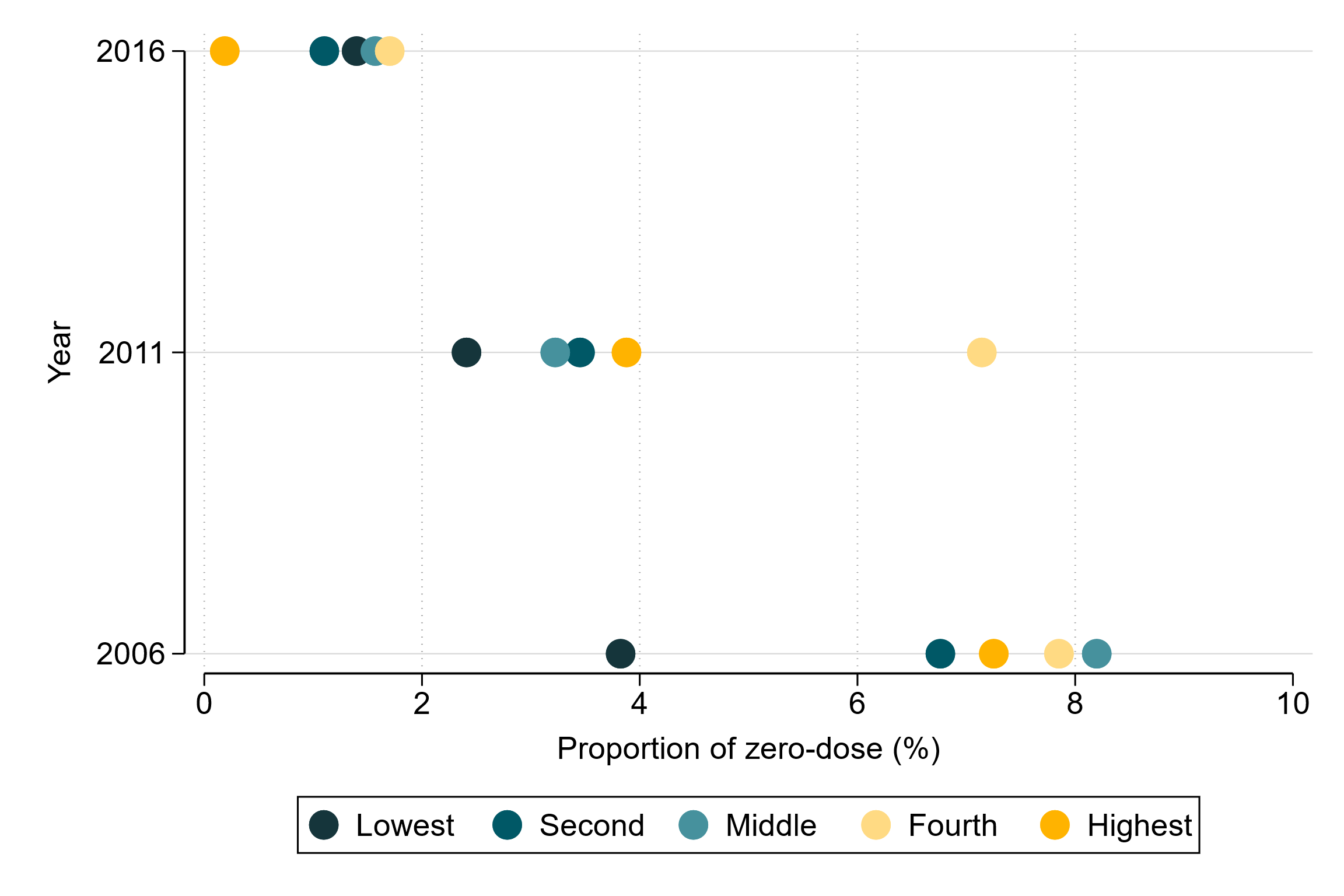

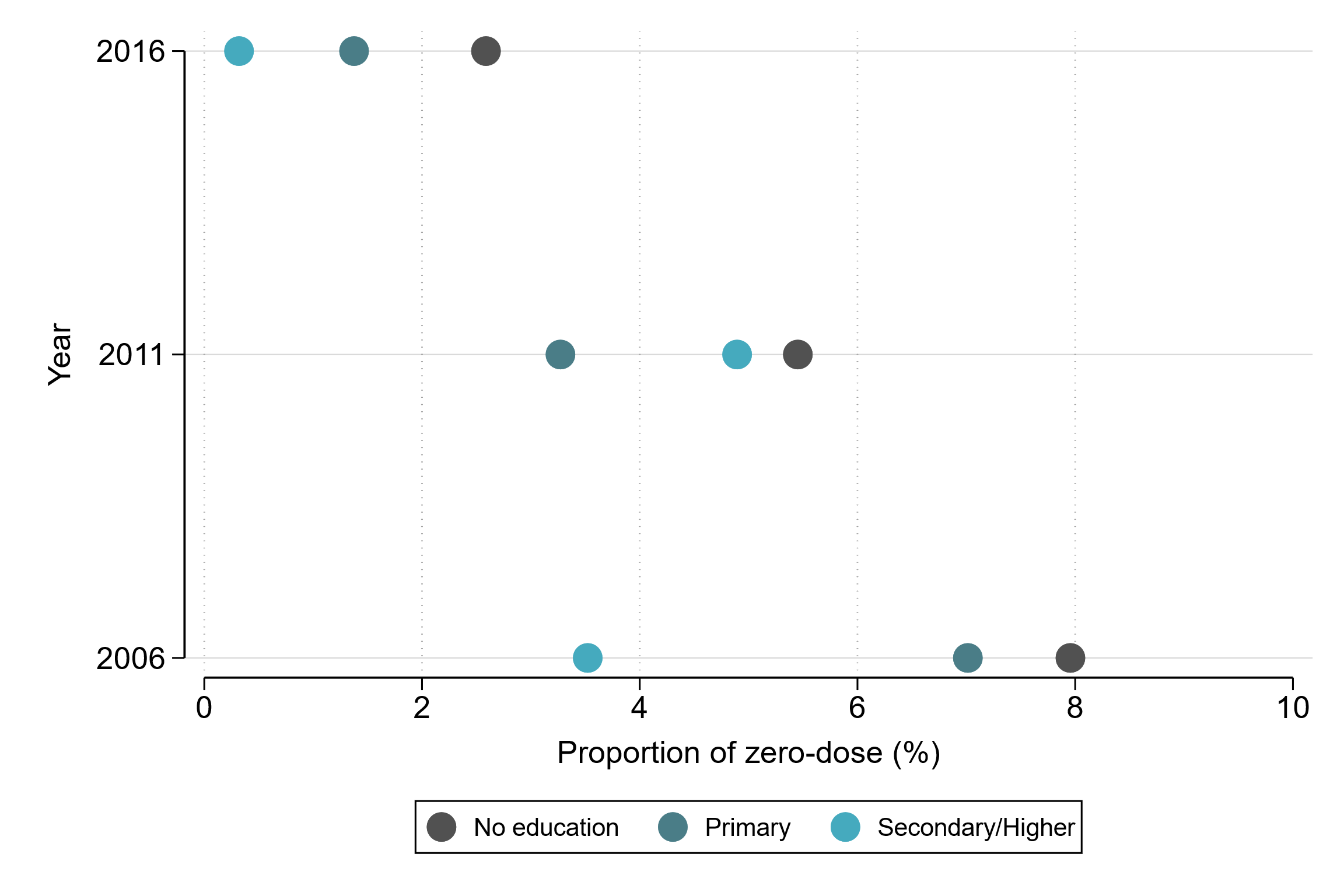


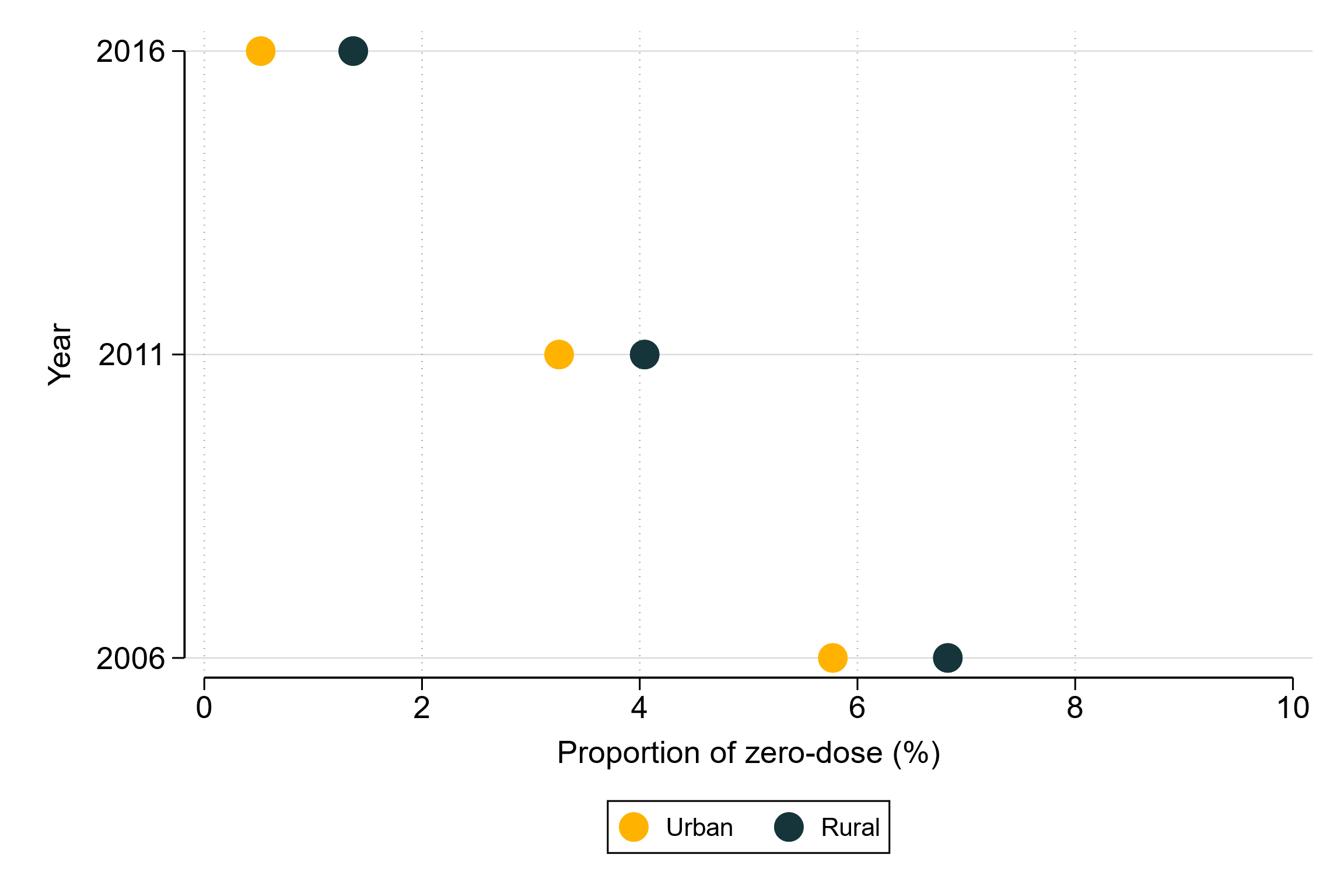


Inequality in the prevalence of zero-dose by wealth quintile, maternal education, and place of residence

Trends in the socioeconomic, and urban-rural inequality in the proportion of under-vaccination among children aged 12-23 months in Uganda

| Dimension of inequality | Year | Difference (%) | Ratio |
| --- | --- | --- | --- |
| Place of residence | 2006 | 4.0 | 1.08 |
|  | 2011 | 10.2 | 1.27 |
|  | 2016 | 1.0 | 1.02 |
| Maternal education | 2006 | 18.5 | 1.45 |
|  | 2011 | 16.6 | 1.44 |
|  | 2016 | 5.4 | 1.15 |
| Wealth index | 2006 | 9.4 | 1.20 |
|  | 2011 | 4.8 | 1.11 |
|  | 2016 | 4.4 | 1.12 |
