# Supplementary material for: Subnational trends and inequalities of under-immunisation and zero-dose among children aged 12–23 months in Uganda: a national population-based cross-sectional study
Source: BMJ Open. 2025 Jan 15;15(1):e093619. doi: 10.1136/bmjopen-2024-093619 (PMC11752051; doi:10.1136/bmjopen-2024-093619)
Supplement: Supplementary file 2 [file bmjopen-2024-093619.reviewer_comments.pdf]

PEER REVIEW HISTORY

BMJ Open publishes all reviews undertaken for accepted manuscripts. Reviewers are asked to complete a checklist review form and are provided with free text boxes to elaborate on their assessment. These free text comments are reproduced below.

ARTICLE DETAILS

Title (Provisional)

Subnational trends and inequalities of under-immunization and zero-dose among children aged 12-23 months in Uganda: a national population-based cross-sectional study

Authors

Wasswa, Ronald; Kananura, Rornald Muhumuza; Waiswa, Peter; Requejo, Jennifer Harris; Santos, Thiago M; Barros, Aluisio JD

VERSION 1 - REVIEW

Reviewer 1

Name McCallum, Alison

Affiliation The University of Edinburgh Usher Institute of Population Health Sciences and Informatics, Centre for Population Health Sciences

Date 15-Oct-2024

COI I chair the Association of Schools of Public Health in the European Region Vaccination Subgroup, commission and contribute to applied research in vaccination, vaccination hesitancy, acceptance and inequalities in coverage and uptake. I am also a member of IANPHI Social and Public Health Inequalities working group and WFPHA NGOs for equity and have advocated for global vaccine equity in the above roles. The positions are unpaid.

Review of immunisation paper

This is a well written paper. It is easy to understand and provides helpful information on trends and inequities in immunisation uptake and coverage in Uganda using a well-respected dataset. It complements other work in this field.

The paper requires some minor revisions. I hope that these will improve it and increase its relevance for users.

Methods

Description of data acquisition and curation by the study team missing

The process for realigning and checking the area of residence is not described in detail. While the description of the process in ref 27 (Belmin, C., Hoffmann, R., Elkasabi, M., & Pichler, P. P. (2022). LivWell: a sub-national Dataset on the Living Conditions of Women and

their Well-being for 52 Countries. Scientific data, 9(1), 719. https://doi.org/10.1038/s41597-022-01824-2) is really clear, this paper would benefit from a sentence or two summarising the process, including the availability of the original data.

The agreed process for minimising the impact of bias and confounding and missing data utilised by the main survey could have been described and the implications for this study stated, including the reasons why there were no issues. Q9 and Q 12 cand g

Note: on checking the survey file, https://www.idhsdata.org/idhs-action/variables/VACMEAS1#codes_section, it is clear that the main survey file for 2016 does not include the variable for 'missing'. There is a variable 'not in the universe' which might have been helpful.

Results

Comparison of study respondents and non-respondents so that the proportions of people in the various categories can be compared with the population of participants. Q13 b and c

Discussion

The findings are promising but it seems likely that they are an underestimate as under and zero vaccination is more likely among children of survey non-participants. I would like to have seen further consideration of the context, scholarship and improvements in practice being undertaken in Uganda and other countries facing similar challenges. Speccifically, this paper complements Ssebagereka, A., de Broucker, G., Ekirapa-Kiracho, E. et al. Equity in vaccine coverage in Uganda from 2000 to 2016: revealing the multifaceted nature of inequity. BMC Public Health 24, 185 (2024). https://doi.org/10.1186/s12889-023-17592-6 and it would have been nice to see where the findings align, provide complementary insights or reasons why they might differ.

It would have been helpful to mention that ref 34 (Hosseinpoor AR, Bergen N, Barros AJ, Wong KL, Boerma T, Victora CG. Monitoring subnational regional inequalities in health: measurement approaches and challenges. Int J Equity Health. 2016;15:18. Published 2016 Jan 28. doi:10.1186/s12939-016-0307-y) highlights the similarity between the findings of more complex analyses and the more straightforward ones undertaken here. This strengthens the case for taking action to address inequities and gaps in coverage based on the analyses in this paper.

At the same time, more detailed studies could be exploring the locally important underlying causes while the implementation of the existing evidence of how to optimise vaccine acceptance is evaluated in partnership with local communities, health professionals and authorities.

Reviewer 2

**Name**	**Fekadu, Hailu**
**Affiliation**	**Arni University, Arsi University**
**Date**	**10-Nov-2024**
**COI**	**'Not applicable'**

Comments reviewer H

In my view the author contribute and learn a lot in throughout the process of identifying inequalities in LMCs. However I have few comments and suggestion for him to increase the readability of the manuscript

1. Why the authors choose only these measure of inequality from the available inequality measures

2. Sex or gender difference in utilization of immunization or vaccination , so why the author did not include this variable

3. Hope, this study conclusion is based on descriptive study, that is diagnostic for the presence of inequality in immunization service utilization. If possible it is good to see determinants of inequality for under utilization

4. The conclusion and recommendation also revised in this line

---

VERSION 1 - AUTHOR RESPONSE

Reviewer 1

We would like to thank the reviewer for the thoughtful and constructive feedback. We have carefully considered all points raised and made revisions to the manuscript accordingly. Below are our responses to each of the comments:

Review of immunisation paper
This is a well written paper. It is easy to understand and provides helpful information on trends and inequities in immunisation uptake and coverage in Uganda using a well-respected dataset. It complements other work in this field.
The paper requires some minor revisions. I hope that these will improve it and increase its relevance for users.
Action: We thank the reviewer for this positive feedback and for recognizing the clarity and relevance of the paper.

Methods
Description of data acquisition and curation by the study team missing

Action: We thank the reviewer for this insightful comment. We have revised the methods section to include a description of how the data was acquired and curated. This additional information can be found on page 5 of the revised manuscript.

The process for realigning and checking the area of residence is not described in detail. While the description of the process in ref 27 (Belmin, C., Hoffmann, R., Elkasabi, M., & Pichler, P. P. (2022). LivWell: a sub-national Dataset on the Living Conditions of Women and their Well-being for 52 Countries. Scientific data, 9(1), 719. https://doi.org/10.1038/s41597-022-01824-2) is really clear, this paper would benefit from a sentence or two summarising the process, including the availability of the original data.

Action: Page 6-We appreciate the reviewer's suggestion. While we refer to the process in Ref 27 (Belmin et al., 2022), we have now added a summary of the harmonization process used to realign and check the area of residence. This includes details on the availability of the original data and the steps taken to ensure accuracy. The updated description is now included in the Methods section.

The agreed process for minimising the impact of bias and confounding and missing data utilised by the main survey could have been described and the implications for this study stated, including the reasons why there were no issues. Q9 and Q 12 cand g
Note: on checking the survey file, https://www.idhsdata.org/idhs-action/variables/VACMEAS1#codes_section, it is clear that the main survey file for 2016 does not include the variable for 'missing'. There is a variable 'not in the universe' which might have been helpful.

Action: We thank the reviewer for this comment. Q9 (efforts to address potential sources of bias): The DHS survey uses a complex sampling design, including stratification and weighting, to ensure that the results are representative at the national and regional levels. Additionally, the DHS methodology aims to minimize non-response and sampling bias. For our study, we used sample weights which account for any non-response and help to ensure representativeness of the sample. However, we also acknowledge the potential for recall bias, which could impact the accuracy of vaccination data, particularly when relying on mothers' memory in the absence of vaccination cards. This limitation has been noted in the manuscript, and Q9 in the STROBE checklist has been updated accordingly.

Q12(c) (missing data): From the DHS, the child's vaccination card is the primary source of data collected on all the vaccines administered and the number of doses. If a vaccination card is unavailable, survey interviewers ask caretakers/mothers about their children's vaccination history, the vaccines administered, and the number of doses received. The possible responses are: Yes, no, or don't know.
In addition, we limited our analysis to children aged 12–23 months which is the relevant population for children to have received immunization, therefore our data set did not encounter missing cases.

Results

Comparison of study respondents and non-respondents so that the proportions of people in the various categories can be compared with the population of participants. Q13 b and c

Action: We appreciate the reviewer's suggestion regarding non-respondents. Since the study used secondary data from the DHS, which employed a nationally representative sampling design, we did not have a separate group of non-respondents to compare to the respondents.

For all the surveys used in these analyses, the response rate was above 95% and thus non-response was minimal. Additionally, the data were adjusted for non-response using sample weights provided by the DHS, which we used in our analysis. As such, the results are representative of the target population, and we have marked items 13(b) and 13(c) on the STROBE checklist as "Not applicable."

Discussion

The findings are promising but it seems likely that they are an underestimate as under and zero vaccination is more likely among children of survey non-participants. I would like to have seen further consideration of the context, scholarship and improvements in practice being undertaken in Uganda and other countries facing similar challenges.

Action: Page 12-14-We thank the reviewer for this suggestion. Our findings are generalizable, and we believe our they reflect the broader context of immunization challenges in Uganda. We have added a discussion on the ongoing government efforts to improve immunization coverage, including the Reach Every District initiative, mass vaccination campaigns, and increased funding for immunization. These efforts have contributed to the progress seen in immunization coverage, though disparities remain, especially in rural areas.

Specifically, this paper complements Ssebagereka, A., de Broucker, G., Ekirapa-Kiracho, E. et al. Equity in vaccine coverage in Uganda from 2000 to 2016: revealing the multifaceted nature of inequity. BMC Public Health 24, 185 (2024). https://doi.org/10.1186/s12889-023-17592-6 and it would have been nice to see where the findings align, provide complementary insights or reasons why they might differ.

Action: Page 12-14-We appreciate the reviewer's suggestion to compare our findings with those of Ssebagereka et al. (2024). Our study aligns with Ssebagereka et al.'s in revealing persistent inequities in vaccine coverage, particularly along socioeconomic and regional lines. We have updated the manuscript to include a more direct comparison with Ssebagereka et al., highlighting where the findings align and providing possible reasons for differences in methodology that could explain some observed discrepancies. Specifically, Ssebagereka et al. defined zero-dose as a child not receiving any vaccines by 12 months, while we included children not receiving any vaccinations between 12-23 months in addition to the measures of inequality adopted. We believe this methodological variation could explain some of the differences in findings.

It would have been helpful to mention that ref 34 (Hosseinpoor AR, Bergen N, Barros AJ, Wong KL, Boerma T, Victora CG. Monitoring subnational regional inequalities in health: measurement approaches and challenges. Int J Equity Health. 2016;15:18. Published 2016 Jan 28. doi:10.1186/s12939-016-0307-y) highlights the similarity between the findings of more complex analyses and the more straightforward ones undertaken here. This strengthens the case for taking action to address inequities and gaps in coverage based on the analyses in this paper.

Action: Page 7: We thank the reviewer's recommendation using Hosseinpoor et al. (2016) to highlight the similarity between the complex and straightforward measures of inequality.

At the same time, more detailed studies could be exploring the locally important underlying causes while the implementation of the existing evidence of how to optimise vaccine acceptance is evaluated in partnership with local communities, health professionals and authorities.

Action: Page 15-We thank the reviewer for this important comment. In the revised manuscript, we have expanded the conclusion section to include a discussion on the need for further research to explore the underlying causes of vaccination disparities, especially in underserved regions. We also highlight the importance of implementing evidence-based strategies in collaboration with local communities, health professionals, and authorities to optimize vaccine acceptance and improve coverage.

Reviewer 2

1. Why the authors choose only these measure of inequality from the available inequality measures.

Action: We thank the reviewer for this insight. We selected these four inequality measures based on their methodological strength and widespread use in prior research. Specifically, the absolute difference and ratio are simple measures that highlight the disparities between the best- and worst-performing groups, allowing for straightforward communication of findings to policymakers. The weighted mean absolute difference from the overall mean (WMADM) and weighted mean ratio from the overall mean (WMRDM) are complex measures that provide a more in-depth understanding by accounting for the population size of each subregion. This ensures that regions with larger populations are weighted appropriately in assessing inequality.

Our choice aligns with recommendations from equity research experts such as Hosseinpoor et al. (34), who emphasize that simple measures often yield results comparable to complex ones while complex measures add depth and context. We therefore aimed for a balance between interpretability and methodological rigor to ensure that our findings are both valid and accessible to a wide audience.

2. Sex or gender difference in utilization of immunization or vaccination , so why the author did not include this variable

Action: We thank the reviewer for this suggestion. We acknowledge the importance of analyzing sex or gender differences in vaccination uptake. However, for this study, our focus was on equity stratifiers that are most commonly used and reported in global health inequality monitoring frameworks, such as regions, maternal education, wealth quintiles, and place of residence (30, 31, 32). These were selected based on their relevance in prior research and their direct implications for targeted programmatic interventions. Additionally, while the UDHS datasets include information on the sex of the child, evidence from Uganda and similar contexts suggests that sex-based disparities in immunization are typically minimal and less pronounced compared to disparities driven by socio-economic and geographic factors. As such, we prioritized variables that have consistently shown significant inequities in vaccination coverage.

We also recognize the potential value of including sex or gender in future analyses and will explore this dimension in subsequent studies to provide a more comprehensive understanding of immunization inequities.

3. Hope, this study conclusion is based on descriptive study, that is diagnostic for the presence of inequality in immunization service utilization. If possible it is good to see determinants of inequality for under utilization.

Action: We appreciate the reviewer's valuable suggestion to explore the determinants of inequality in immunization utilization. Our study primarily aimed to describe and quantify inequalities in under-vaccination and zero-dose vaccination across various equity dimensions, offering a diagnostic overview of where disparities exist.

As noted in our introduction, the determinants of under-immunization—such as socioeconomic status, maternal education, and geographic location—are known and well-documented in the literature. This study was designed to complement that body of work by specifically identifying who is left behind in the pursuit of equitable immunization coverage.

While this study is primarily descriptive, its contribution lies in emphasizing the populations being left behind and advocating for urgent, targeted actions to address these inequities. Understanding the gap between the most advantaged and the least privileged populations provides actionable insights for policymakers and health planners.

However, future studies could build on this foundation by investigating specific determinants of these inequalities in greater depth, leveraging inferential analyses to uncover causal pathways and inform strategic interventions.

4. The conclusion and recommendation also revised in this line

Action: We thank the reviewer for their thoughtful suggestion. However, our conclusion and recommendations are directly informed by the study findings, which are aligned with the study objectives.
